# Small Extracellular Vesicles from Human Amniotic Fluid Samples as Promising Theranostics

**DOI:** 10.3390/ijms23020590

**Published:** 2022-01-06

**Authors:** Ambra Costa, Rodolfo Quarto, Sveva Bollini

**Affiliations:** 1 Experimental Biology Unit, Department of Experimental Medicine (DIMES), University of Genova, 16132 Genova, Italy; ambra.costa@edu.unige.it (A.C.); rodolfo.quarto@unige.it (R.Q.); 2 Cellular Oncology Unit, IRCCS Ospedale Policlinico San Martino, 16132 Genova, Italy

**Keywords:** amniotic fluid, stem cells, extracellular vesicles, exosomes, regenerative medicine

## Abstract

Since the first evidence that stem cells can provide pro-resolving effects via paracrine secretion of soluble factors, growing interest has been addressed to define the most ideal cell source for clinical translation. Leftover or clinical waste samples of human amniotic fluid obtained following prenatal screening, clinical intervention, or during scheduled caesarean section (C-section) delivery at term have been recently considered an appealing source of mesenchymal progenitors with peculiar regenerative capacity. Human amniotic fluid stem cells (hAFSC) have been demonstrated to support tissue recovery in several preclinical models of disease by exerting paracrine proliferative, anti-inflammatory and regenerative influence. Small extracellular vesicles (EVs) concentrated from the hAFSC secretome (the total soluble trophic factors secreted in the cell-conditioned medium, hAFSC-CM) recapitulate most of the beneficial cell effects. Independent studies in preclinical models of either adult disorders or severe diseases in newborns have suggested a regenerative role of hAFSC-EVs. EVs can be eventually concentrated from amniotic fluid (hAF) to offer useful prenatal information, as recently suggested. In this review, we focus on the most significant aspects of EVs obtained from either hAFSC and hAF and consider the current challenges for their clinical translation, including isolation, characterization and quantification methods.

## 1. Introduction: Human Amniotic Fluid Stem Cells as Reservoir of Paracrine Factors

Mesenchymal stromal cells (MSC) are progenitor cells that can be isolated from several tissues obtained from both adult and perinatal foetal tissue (i.e., bone marrow, adipose tissue, cord blood, placenta, amniotic fluid, etc.) [1]. In this scenario, human amniotic fluid (hAF)-derived stem cells are immature MSC that can be easily obtained from extra-embryonic annexes during gestation; indeed hAF-MSC can be easily isolated as foetal stromal progenitor cells from leftover samples of routine prenatal screening (i.e., II trimester amniocentesis amniotic fluid sampling) or as perinatal progenitors at birth from clinical waste (i.e., III trimester term amniotic fluid obtained during scheduled Caesarean delivery) [2,3,4,5,6,7,8,9,10,11]. hAF-MSC have been described as multipotent cells that can be extensively and easily expanded and cryopreserved with stable karyotype due to their remarkable self-renewal profile, while not being tumorigenic and lacking ethical concerns [2,12,13,14]. These stem cells have been described as a heterogeneous population and, according to their morphology, they can be classified into (i) epithelioid cells, also defined as round-shaped (RS), or (ii) fibroblastic ones, namely spindle-shaped (SS) [15,16]. Unravelling such heterogeneity may offer important information on the stemness and multipotency of these progenitor cells, as discussed in [6,17]. Indeed, to date, a general consensus has not yet been reached on a standardised protocol to isolate a more homogeneous population of human amniotic fluid progenitors. While some researchers have described the regenerative potential of RS- or SS-hAF-MSC obtained as the adherent stromal population retrieved from amniotic fluid [16,18,19,20], the enrichment of specific subpopulation, based on the specific expression of the stem cell factor c-KIT (CD117), has been reported to identify stem cells within the human amniotic fluid which may harbour the highest self-renewal potency, namely c-KIT+ human amniotic fluid-derived stem cells (hAFSC) [2,3]. In these last 20 years, hAFSC have gained increasing attention as appealing source of immature stem cells that are multipotent, possess immunomodulatory properties and do not have the ethical and legal limitations of embryonic stem cells, thus being extensively investigated for tissue engineering applications as well as in transplantation strategies for the treatment of various degenerative and inflammatory diseases affecting major tissues/organ in either neonatal/paediatric patients and adult ones [12,21].

Notably, several independent studies in the field of regenerative medicine have highlighted that the therapeutic potential of transplanted stem cells cannot be provided by their integration and differentiation in the host tissue, but rather to the release of secreted soluble trophic factors orchestrating a pro-resolving microenvironment within the injured tissue [20,22,23,24,25,26,27]. As a matter of fact, in the last 15 years, the so-called *stem cell-paracrine effect* has significantly shifted the initial attention on the progenitor cell genome towards a more accurate characterization of their secretome (i.e., the heterogeneous set of trophic molecules, including individual factors and membrane-bound extracellular vesicles released by cells), as an appealing therapeutic tool. In such perspective, the MSC and stem cell secretome has been widely investigated to provide proof-of-principle paracrine therapy to restore and enhance the endogenous mechanism of repair in injured tissues and organs, following hypoxic, oxidative, inflammatory and necrotic injuries, in order to provide a cell-free therapeutic approach. A growing body of studies has further specifically focused on the particulate fraction within the stem/progenitor cell secretome, namely their extracellular vesicles (EVs), as relevant biological conveyors of tissue restoration and regeneration [28]. In fact, EVs are well known to mediate paracrine inter-cellular communication, so as to have a critical impact on physiological and pathological conditions [29,30].

EVs can be isolated from cell-conditioned medium, as well as from several body fluids (i.e., plasma, serum, synovial fluid, cerebrospinal fluid, seminal fluid, etc.) [31,32] as heterogeneous micro- and nano-scaled particles enclosed by a lipid envelope, with size ranging from 50 nm up to 1 µm. According to the minimal guidelines of the International Society of Extracellular Vesicles (MISEV2018), EVs can be defined according to their size or density, biochemical composition of their content, and their specific (cell) source conditions. They can be generally subdivided into small EVs (sEVs, <200 nm, also referred to as exosomes) and medium and large EVs (also defines as microvesicles, mEVs and lEVs, >200 nm) [33]. Thus far, this definition helps to describe their biogenesis: small EVs originate from intracellular late endosomes or multivesicular bodies (MVBs), previously produced by endo-lysosomal system, which, upon fusion to the cell membrane, delivers them in the extracellular microenvironment. Microvesicles and larger EVs are released by means of budding of the plasma membrane [34]. The EV content and phenotype is largely outlined by proteins involved in their biogenesis (i.e., tetraspanins, flotillins and heat shock protein) and primed by the cell and/or tissue they originate from [35,36]. Indeed, EV release is activated as a response to the specific stimuli the secreting parental cell may experience in terms of exogenous stress or preconditioning [37]. Small EVs can also shuttle between cells genomic and mitochondrial DNA (gDNA and mtDNA) [38], messenger RNA (mRNA), microRNA (miRNA) and long non-coding RNA (lncRNA) along with bioactive factors, such as proteins and lipids [39,40]. Notably, EVs have been shown to influence several biological processes, such as angiogenesis [41], cell migration [42], regulation of inflammation [43], tissue repair and regeneration [44,45] due to their paracrine cargo. Thus, stem cell/progenitor cell-derived EVs may be envisioned as attractive off-the-shelf medicinal therapeutic products for future cell-free paracrine therapy, also in light of their putative low immunogenicity.

Since hAF-MSC and hAFSC represent a developmentally immature and more juvenile cell source than adult somatic MSC, they can represent an ideal supply of small EVs endowed with functional pro-resolving potential. Moreover, they can offer an optimal exploitable choice given their ease of isolation and remarkable self-renewal potential. Indeed, EVs concentrated from the human amniotic fluid progenitor secretome have been attracting increasing interest lately; the EV yield obtained from human hAF-MSC has been proven to be higher than human bone marrow-MSC [46]. Several independent studies have reported that the hAF-MSC and hAFSC secretome exert pro-survival, anti-apoptotic effects [47], while quenching inflammation [48], stimulating local angiogenesis [49,50], and supporting cardiac protection following myocardial infarction or cardiotoxicity [49,51,52,53]. In this scenario, hAFSC-EVs have been widely profiled to assess whether they can recapitulate such pro-regenerative potential with promising results in preclinical models of skeletal and cardiac muscle injury [19,47,49,54], lung [55] and neurodegenerative disease [56,57], osteoarthritis [58], osteoporosis [59] and cutaneous injury [60].

Notably, small EVs have been recently separated and concentrated directly from human amniotic fluid samples for biomarker discovery and to provide relevant diagnostic tools to assess molecular alteration of the foetal status, as well as serving as a predictor of ongoing pregnancy and preterm labour [61,62,63]. Nevertheless, a very recent study has also indicated that term hAF-EVs may exert therapeutic effects, since modulating inflammation while counteracting oxidative stress in a preclinical model of experimental bronchopulmonary dysplasia [64]; a case report on the administration of a novel formulation of small hAF-EVs in three critically ill patients suffering from severe COVID-19 disease has also demonstrated their safety and feasibility for respiratory failure induced by COVID-19 infection.

In this comprehensive review we will: (i) discuss the role of hAF-MSC/hAFSC-EVs (here collectively referred to as hAFS-EVs), as promising therapeutics for paediatric and adult disease; (ii) illustrate their characterization by comparing the current and most up-to-date methods and technical challenges, and (iii) eventually consider the key role of hAF-EVs as candidate theranostic tools.

## 2. EVs from Human Amniotic Fluid Stem Cells as Promising Medicinal Therapeutics

To the best of our knowledge, the first study demonstrating the therapeutic effect of hAF-MSC-EVs dates back to 2012 [65], as showing that microvesicles from human amniotic fluid-derived mesenchymal stromal cells reduced pathological cystine accumulation in cystinosin (CTNS)-knock out fibroblasts in vitro, by decreasing their levels of mutant protein. Since then, the interest in the paracrine potential of hAFS-EVs has significantly increased in preclinical translational research for regenerative medicine. From such a perspective, EVs from human amniotic fluid stem cells have been assessed as promising therapeutics for either paediatric disease and adult injuries.

### 2.1. hAFS-EVs as Advanced Medicinal Therapy Products for Prenatal and Neonatal Disease

Since hAF-MSC and hAFSC can be easily obtained, cryopreserved and banked, they offer an ideal cell source to obtain small EVs to be further stored and envisioned as ready-to-use formulations for paracrine therapy whenever needed, such as for newborn/neonatal patients in demand of prompt intervention at birth/soon after birth. Indeed, independent studies have been lately developed to assess the therapeutic regenerative potential of hAFS-EVs for severe paediatric disease and malformations.

Necrotizing enterocolitis (NEC) is one of the most dangerous diseases in premature infants. To date, the therapeutic approach is surgical care, with a mortality rate of around 50% [66]. In addition, surviving infants present several medical complications, thus alternative therapeutic approaches are strongly required [67]. In particular, NEC may arise as a consequence of intestinal stem cell (ISC) loss, following defective Wnt signalling [68]; in physiological conditions, ISC mediates the intestinal regeneration after injury, but chronic damage of the gut affects ISC with additional worsening caused by prolonged inflammation. A pivotal study in 2014 reported that rat AFSC improved survival and enhanced repair of the damaged intestine in a preclinical rodent NEC model via a paracrine COX-2 (CycloOxygenase-2)-dependent mechanism by decreasing apoptosis and mucosal inflammation [48]. More recently, follow-up studies demonstrated that either rat- or human AFSC-EVs could recapitulate such therapeutic effects [67,68]. Indeed, authors reported that AFSC-EVs counteracted intestinal injury by restoring the Wnt pathway [68]. Notably, intraperitoneal injection of 100 µL hAFSC-EVs (released from about 6 × 10^6^ cells) in neonatal mice decreased tissue damage in the terminal ileum, the region affected mostly by NEC. Moreover, the EV treatment resulted in a 1.8-fold increase in Ki67 expression in the terminal ileum, suggesting tissue regeneration due to endogenous progenitor proliferation, as also suggested by upregulation of the specific intestinal stem cells marker gene (leucine-rich repeat-containing G-protein coupled receptor 5, *Lgrp5*). hAFSC-EV treatment quenched inflammation, a hallmark of the NEC mouse model as well as of affected infants, by normalizing IL-6 and TNF-α levels [67].

Another remarkable therapeutic application of hAFSC-EVs has been recently reported by Antounians et al. [55], as potential future paracrine therapy for congenital diaphragmatic hernia (CDH). During embryogenesis, defective lung development can result in pulmonary hypoplasia, characterised by less bronchiolar divisions, defective alveolarization, and reduced tissue maturation [69]. The most frequently developmental alteration leading to pulmonary hypoplasia is CDH. Newborns suffering from CDH present an underdeveloped diaphragm with herniation of intra-abdominal organs into the chest [69,70]. The mortality rate in the very first days can reach up to 40% [71] with the remaining 60% affected by several morbidities [72]. The promising role for small AFS-EVs in this pathological setting was suggested by previous independent evidence on the paracrine effects by AFSC and AF-MSC in reducing lung fibrosis, supporting alveolar epithelial cells after injury and increasing the lung growth in a model of hypoplasia secondary to CDH [73,74,75,76,77]. Using foetal rodent models of nitrofen-derived pulmonary hypoplasia (including organoid studies), Antounians et al. assessed rat AFSC-EVs as regenerative therapeutics over the corresponding total secretome, resulting in an increase in terminal bud count and lung surface area; interestingly, authors reported that such a therapeutic role was dose dependent and also size dependent: only small rAFSC-EVs (~140 nm) exerted this beneficial effect. Notably, to validate in vivo rAFSC-EVs, a surgical model of pulmonary hypoplasia secondary to CDH was optimised in foetal rabbits in order to provide proof-of-principle for future in utero therapy with EV local administration. rAFSC-EVs were confirmed to improve lung alveolarization and promoted alveolar lipofibroblast maturation [55]. Since micro RNAs (miRNAs) have been shown to be relevant in lung development and their dysregulation can induce hypoplastic lung, therefore, against such a scenario, AFS-EVs may be envisioned as therapeutic conveyors of critical RNA content. Indeed, rAFSC-EVs were found to be enriched in the miR17-92 family, involved in lung development, along with high expression of BMP signalling, critical for alveolar maturation. Likewise, GMP (good manufacturing practices)-compliant hAFSC-EVs replicated the same beneficial results both in vitro and in vivo in the preclinical rabbit model, demonstrating superior efficacy over human bone marrow-MSC-EVs. In light of these results, hAFSC-EVs may be considered promising therapeutics for prompt prenatal treatment of foetal congenital disease at the time of diagnosis, with tremendous potential to prolong the life expectancy of babies affected by severe and fatal diseases.

### 2.2. Small hAFS-EVs as Therapeutics for Adult Disease 

The translational potential of hAFS-EVs has also been also largely investigated in several preclinical models of adult ischemic- or inflammatory-based disease.

The rationale comes from studies demonstrating that systemic injection of hAFSC-conditioned medium can provide prompt cardioprotective effects, by decreasing infarct size and enhancing myocardial cell survival in a rat model of acute ischaemia-reperfusion injury [53]. Similar cardioprotective and stimulatory paracrine effects from the secretome of human amniotic fluid progenitors were also obtained by follow-up studies based on models of doxorubicin-induced cardiotoxicity both in vitro and in vivo [51,52] and from independent work in a myocardial ischemia-reperfusion (I/R) injury mouse model [78]. In particular, myocardial infarction (MI) can often lead to the onset of heart failure (HF), which afflicts up to 38 million people in the world [79]. In order to enhance endogenous, yet defective, cardiac repair mechanism which is activated following MI and can lead to detrimental remodelling and scarring, innovative therapeutic strategies are under investigation. Indeed, efficient cardio-protection, improvements in neovascularisation, modulation of inflammation and myocardial renewal represent critical aspects that EV-based paracrine therapy may target. Our group reported that a single intra-myocardial injection of 4.5 µg hAFSC-EVs in mice in the acute setting following MI results in prolonged cardio-active effects with improvement in cardiac function up to 1 month from treatment [49]. Moreover, the injected small EVs increased bromodeoxyuridine (BrdU) uptake in resident surviving cardiomyocytes, suggesting their activation towards cell cycle re-entry, along with the awakening of cardiac progenitor cells from the epicardium, a source of regenerative paracrine potential in the adult heart after injury [80,81]. Small hAFSC-EVs did not influence local angiogenesis in the injured cardiac tissue, in contrast to the total secretome originating from hAFSC-CM, suggesting that such a role may be rather conferred to other soluble factors. In a similar study, Takov et al. reported that 1.7 × 10^10^ particles/μg protein of small hAF-MSC-EVs (SS-hAF-MSC-EVs) significantly decreased infarct size in a preclinical rat model of myocardial ischaemia/reperfusion (I/R) injury when administered intravenously prior to reperfusion. Notably, while effective in vivo, such EV formulation did not exert relevant pro-survival effects on isolated primary cardiomyocytes in vitro, suggesting that their cardioprotective potential could be exerted via an indirect mechanism of action; similar to hAFSC-EVs, these hAF-MSC-EVs were not proangiogenic on cardiovascular cells, yet they stimulated endothelial progenitor migration [19]. In a rat model of isoproterenol (ISO)-induced cardiac fibrosis, small hAF-MSC-EV treatment concurred to decrease the expression of pro-fibrotic markers, while increasing micro-vessel density within the myocardium [82]. hAFSC have also been described to be effective in counteracting and delaying the progression of fibrosis in the kidneys of preclinical animal models of Alport Syndrome; thus, hAFSC-EVs were tested to assess whether they could be responsible for such renoprotection. In fact, EVs (2 × 10^11^ particles as produced by 1 × 10^6^ cells) were shown to work as a decoy to modulate VEGF (vascular endothelial growth factor) in glomerular endothelial cells, by binding the excess VEGF through, thus preventing cellular damage. These hAFSC-EVs expressed VEGFR1 and VEGFR2 on their surface, while they did not contain VEGF as part of their cargo; yet, they could specifically modulate VEGF/VEGFRs signalling due to their miRNA content including miR-161, miR-23a, miR-27a, miR-93, miR-221, miR-145 and miR-322 [83]. Altogether, these results suggest that hAFS-EVs have relevant cardio- and reno-protective effects over vascular ones, although it is not clear whether the mechanism of action may be influenced by their dose, administration route, or specific cell subtype origin.

hAFSC-EVs have been reported to deliver regenerative paracrine influence in the skeletal muscle tissue, according to recent work employing either a double knock-in transgenic murine model mimicking skeletal muscle atrophy [47] or a cardiotoxin-induced injury of the tibilialis anterior (TA) muscle [54]. While the two studies employed different doses and delivery systems (intra-muscular injection of 1 µg of hAFSC-EVs in the hind limb [47] versus systemic delivery via tail vein of EVs within 100 µL of hAFSC-CM in PBS solution), results were coherent in sustaining survival, while promoting anti-inflammatory effects. In the genetically-induced chronic in vivo model of muscle dysfunction, hAFSC-EVs swiftly modulated inflammation and pro-resolving chemokines (IL-10), while inducing pro-resolving phenotype in infiltrating macrophages (M2) over the pro-inflammatory ones (M1) [47]. These restorative effects were lost within a few days, suggesting the therapeutic need for prolonged and sustained administration over time. In the acute setting of cardiotoxin-induced injury, hAFSC-EVs also delivered muscle regeneration and increased vessel density, with putative molecular candidates within their cargo, including miRNAs (let7b-5p; miR382; miR-24-3p; miR3960), identified capable of modulating proliferation, inflammation and angiogenesis [54].

The immunomodulatory effect of hAF-MSC-EVs has been reported to be related to IFNγ (interferon-gamma) priming of the parental secreting cells [84]. In particular, the immunomodulatory paracrine potential of hAF-MSC seems to be mediated by indoleamine 2,3-dioxygenase 1 (IDO1); this is further enhanced when cells are stimulated by IFNγ. IDO1 is known to regulate functions in autoimmune [85] and inflammatory response [86]. Notably, a high level of IDO1 in SS-hAF-MSC could be transferred into their secreted EVs; thus, hAFS-EVs prolonged skin engraftment in vivo, likely due to their immunomodulatory function, increased the subpopulation of regulatory CD4^+^CD25^+^ T-cells and decreased T-cell proliferation. hAFSC-EVs have been described to be endowed with immunomodulatory functions even without prior cell priming [87]; PBMC (peripheral blood mononuclear cells) treated with hAFSC-EVs in vitro (80 µg of EVs for 1 × 10^6^ target cells) showed a decreased amount of CD4+ T-helper cells with a low level of BrdU uptake, suggesting that EVs inhibited lymphocyte proliferation, coherently with previous work. The regenerative role of small hAFSC-EVs has also been tested against a monoiodoacetate-induced preclinical rat model of osteoarthritis. Rats that were administered 100 µg of HGF (hepatocyte growth factor), TGF-β (transforming growth factor-beta), and IDO-enriched EVs in the knee joints with follow-up boost every 10 days until 6 weeks, presented cartilage restoration and fibrosis reduction. Moreover, cells expressing macrophage M2 markers were detected in the treated joints, indicating translational efficacy of hAFSC-EVs in treating cartilage damage, by means of their TGF-β content [58].

Topic administration with 20 µg hAFSC-EVs once a week improved also wound healing in a preclinical rat model of cutaneous injury [60]. After 28 days, EVs treatment resulted in increased angiogenesis by a high level of CD31 expression, with inhibition of fibrotic scarring. Prolonged activation of myofibroblast is positively regulated by TGF-β family element, via SMAD protein phosphorylation [88]; EV-induced cutaneous regeneration has been suggested to be mediated by their miRNA (miR-27a, miR23a, miR22, miR-21, let-7-5p) content inhibiting the *TGF-β* receptor mRNA, thus impacting scar formation. Therefore, hAFS-EVs are likely to exert their beneficial pro-resolving paracrine effect via a putatively stable miRNA core in their cargo, as the ones identified in this work have also been reported in other applications [47,54,89].

hAF-MSC and hAFSC paracrine effects have been also broadly tested in models of neurovascular and neurodegenerative diseases. hAF-MSC-EVs were demonstrated to deliver significant neuroprotection through anti-apoptotic and pro-survival pathways modulated by their miRNA content in an in vitro ischemia/reperfusion (I/R) and oxygen and glucose deprivation stroke model on a neuron-like cell line [56]. Here as well, some of the most relevant miRNA described as a molecular signature of hAF-EVs have been reported in other independent studies as well (i.e., miR146a, miR29a, miR27a, miR221 [47,54,89]). Alzheimer’s disease (AD) is characterised by increased oxidative stress; given the remarkable pro-survival and antioxidant paracrine potential of hAF-MSC and hAFSC, it may be reasonable to envision hAFS-EVs as candidate therapeutics in this field. Indeed, hAFSC-EVs (as a 10 μg/10^6^ target cell dose), counteracted the in vitro disease phenotype in AD neuron primary culture. In particular, compromised cells treated with EVs showed increased numbers, length, and diameter of their neurites, with reduced production of reactive oxygen species (ROS), NADPH oxidase 4 (NOX4) while enhancing anti-oxidative enzymes (i.e., superoxide dismutase 1, SOD1, thioredoxin reductase 1, TrxR1, thioredoxin reductase 2, TrxR2, glutathione peroxidase, Gprx1) and decreasing amyloid-beta (Aβ) and Tau protein expression [57].

Further studies are in support of the anti-oxidative potential of hAFS-EVs in a model of steroid-induced osteoporosis; osteoblasts treated with dexamethasone (which mimics in vitro osteoporosis damage) and receiving hAFS-EVs showed activation of survival signals, inhibition of cell death pathways, via Akt signalling, osteogenic differentiation, low ROS levels and increased the expression of anti-oxidative enzymes [59].

## 3. From the Bench to the Bedside: Translating Promising Results over Methodological Concerns

Given the results reported by several independent lines of investigation, hAFS-EVs may represent an appealing source of therapeutics for several diseases. Yet, there is a strong need for defining proper standardization in their isolation, quantification and application in order to define a reliable reference system and to be able to compare different studies and their translational potential. Here we will consider the most relevant technical aspects which may influence hAFS-EV biological activity and future clinical translation for paracrine therapy.

### 3.1. Tuning Human Amniotic Fluid-Stem Cell Secretory Activity to Separate and Concentrate EVs

The whole of stem cell therapeutic factors which are secreted during their in vitro culture, including both soluble factors (i.e., cytokines, chemokines, proteins, etc.) and EVs, is represented by the cell-conditioned medium (CM). Thus, stem cell-EVs can be separated starting from cell-CM and further concentrated by different methods. From this perspective, cell culture condition represents a key aspect that can be finely tuned to prime cells to enrich their CM with bioactive molecules and EVs. 

Among the variables which may mostly influence progenitor cell secretome and the yield and purity of their EVs, there is the need of removing foetal bovine serum (FBS) from the culture medium, to avoid any contamination from FBS-related factors. This can be easily obtained by either incubating cells in xeno- and serum-free medium (SF) formulations or by employing commercially available or home-made FBS-EV-free supplements. Most studies focusing on hAFSC-EVs apply SF culture conditions [19,46,47,54,57,58,60,67,84,87,89], while fewer groups have reported using EV-free culture medium to collect the cell-CM [55,56]. Likewise, other variables have been shown to prime stem cell and MSC secretory potential, such as oxygen tension or pro-inflammatory stimuli (i.e., IFNγ) [84]. Indeed, several groups reported that in vitro hypoxic preconditioning may lead to an increased secretome yield, including EV fractions [47,54,89]. The duration of preconditioning is another sensible aspect, since hAF-MSC/hAFSC-CM has been reported to be collected after 24 h [46,47,67,89], 48 h [19,60], 72 h [56] or 96 h [57,58]. Notably, hAF-MSC and hAFSC have been suggested to mature different miRNA profiles which can be further enriched in their EV cargo, depending on culture conditions [54]. Remarkably, hAFS-EVs should be produced in GMP setting and from hAFSC stimulated/primed under conditions compliant with clinical translation. This would ensure a biologically active secretome, yet free of any xeno- and exogenous molecules, as suggested by Mellows et al. when using PBS (phosphate-buffered saline) solution to obtain hAFSC-EVs [54].

Being human amniotic fluid stem cells a heterogeneous population, the protocol used for their isolation may also represent an additional aspect to be taken into consideration. It is reasonable to consider that subpopulation of human amniotic fluid progenitors may release different paracrine factors depending on their selection protocol (i.e., c-KIT+ hAFSC over hAF-MSC) and culture condition. In addition, quite different culture media have been reported to be used for the in vitro subculture of either hAFSC (i.e., with the specific use of Chang medium as originally according to De Coppi et al. [2,47,49,51,53,58,59,87,89,90]), or hAF-MSC [19,20,84,87]. Thus, there is an urgent need for standardization of the human amniotic fluid progenitor culture conditions and the preconditioning protocols to prime their secretome and EVs.

### 3.2. Looking for Consensus on Small hAFS-EV Isolation Methods

Several techniques have been reported to separate and isolate EVs from hAF-MSC and hAFSC secretome, as addressed below. Each specific method may present specific advantages and limits, as summarised in Table 1.

#### 3.2.1. Differential Ultracentrifugation (dUC) and Density Gradient Ultracentrifugation

Serial differential ultracentrifugation (dUC) is the most frequently used method to obtain small EVs from cell-conditioned medium [91,92]. This method allows removing cells, cellular debris, and putative apoptotic bodies [91] resulting in a pellet of small EVs following several rounds of centrifugal precipitation at remarkably high speed. Indeed, the *g* acceleration applied, together with the viscosity of the sample, determines EV separation efficiency [44]. Thus, dUC permits obtaining either medium-sized EV or microvesicles (mEVs), as the pellet obtained after the 10,000× *g* run, and sEVs, which precipitate following the 100,000× *g* run. Specifically, two different rotors can be employed to isolate the EV fraction: a swinging (SW) or fixed-angle (FA) rotor, with different outcomes: SW rotor can result in superior purity but with a lower yield of EVs, compared to the FA one. Since dUC is influenced by several technical aspects (acceleration, length of the run, sample viscosity and type of rotor), the overall results may be inconsistent, while being quite a time-consuming method [44]. Yet, this is a low-cost procedure and it can be used to further concentrate EVs downstream other isolation methods (such as size exclusion chromatography, as discussed later).

Most studies addressing the regenerative potential of EV fractions obtained from human amniotic fluid progenitor-conditioned medium are based on dUC as the main method [47,54,55,56,60,84,89]. In particular, while different dUC protocols are reported in the literature to concentrate these perinatal EVs according to the length of the run and acceleration; there is only one study that has reported the use of FA rotors [54]. 

The density gradient ultracentrifugation technique exploits a high-speed centrifuge with different floating gradients to isolate EVs in solution. The use of the gradient solutions (such as sucrose or, more frequently, iodixanol, also known as Optiprep) allows to build up a gradually decreasing density from the bottom to the top of the test tube in order to separate EVs depending on size, mass and density [93]. This process results in a relatively high purity of EVs, which result clearly separated from protein contaminants. However, density gradient UC is time-consuming too as requiring additional steps to carefully wash the EV pellet. Indeed, remnants of density solution can stick to the EV surface, thus influencing their therapeutic activity and affecting downstream characterization analyses, as for example RNA sequencing [44]. To date, density gradient ultracentrifuge (with sucrose) has also been reported to isolate hAF-MSC-EVs in combination with dUC [84].

#### 3.2.2. Ultrafiltration

Ultrafiltration is based on specific size discrimination of EVs, by means of membrane filters with pores of different dimensions: pores are designed to be smaller than EVs subfractions so that bigger particles are eluted and the EVs become enriched on the membrane. This procedure allows very rapid, reproducible, and relatively low-cost separation of EVs; in addition, it can be employed to concentrate EVs (such as dUC, but by less time-consuming procedure), downstream other isolation methods. Yet, the pressure exerted by external centrifuge applied to the sample may affect EV morphology [94]. To avoid this problem, ultrafiltration with a tangentially applied flow has been developed, namely tangential flow filtration (TFF), allowing the separation between proteins and EVs [95]. Ultrafiltration has not been reported yet for isolating hAFS-EVs.

#### 3.2.3. Poly-Ethylene Glycol (PEG)

Poly-ethylene glycol (PEG) isolation of EVs is based on precipitation technique. The PEG is an aqueous solution which aggregates with EVs and, thus, determines their precipitation at low-speed centrifugation (1500× *g*) [96]. This method is user friendly and the technique is easily made available by commercial kits which utilise the PEG-based precipitation (such as ExoQuick and Total Exosome Isolation Reagent Kit, TEIR). Yet, protein contamination as co-precipitating with EVs may occur, thus affecting data interpretation. PEG-based precipitation method is one of the most commonly utilised to isolate the EV fractions from the conditioned medium released by hAFS [46,57,58,59,87]. Of interest, when hAFSC-EV yield and activity were characterised according to these different protocols for processing hAFSC-CM (ExoQuick, TEIR and dUC), no major differences were found in the EV preparation obtained by ExoQuick and dUC in terms of yield and biological function; in contrast, hAFSC-EVs isolated by TEIR presented a lower yield and efficacy [97]. These results suggest that, although the same PEG methodology was applied, results may vary, thus questioning the consistency of the technique. Nonetheless, independent evidence has indicated that PEG-based EV isolation may better preserve vesicle surface; in particular, PEG may protect proteins on EV surface when compared to dUC, thus possibly better preserving their biological potential [98]. Being a quite simple and straightforward procedure, the PEG-based precipitation method, together with dUC, is the most commonly used technique documented for hAFS-EV separation; moreover, the hAFS-EVs obtained showed a very high yield [46,57,87], although protein contamination affecting this evaluation may not be excluded.

#### 3.2.4. Size-Exclusion Chromatography (SEC)

Size-exclusion liquid chromatography (SEC) is one of the main chromatographic techniques used for the isolation of EVs to separate them in solution based on size. SEC is performed by means of a porous solid matrix in a column through which the sample is eluted. This procedure allows to first elute proteins, which are smaller than EVs and thus pass the column more rapidly, and then the EV fractions [99]. The interest in this application in the EV field is growing due to its efficiency, reproducibility, very low protein contamination and purity of EV yield, which makes it also ideal to isolate EVs from biological fluids [100]. Compared to ultrafiltration, there are a few studies based on SEC to obtain hAFS-EVs [19,46]. Interestingly, Tracy et al. applied TEIR alone and in combination with SEC to compare the size distribution, morphology, expression of typical surface markers, and yield of small hAFS-EVs versus human bone marrow-MSC ones; they reported that, following SEC, EV size diminished with a 10-fold increase in very small EVs (<102 nm) [46].

#### 3.2.5. Anion-Exchange Chromatography

Anion-exchange (AEX) chromatography is based on exploiting ion charge. A stationary phase is enclosed into a column possessing a cationic exchange, thus binding any anionic/negative component. When the sample enriched in EVs is eluted through the column, the stationary (cationic) phase binds the EVs and the other components get washed away. Thus, proteins and other small particles are eluted before EVs [101]. AEX has the potential to become a reproducible and automatized procedure by using fast protein liquid chromatography (FPLC) [101]. This method exploits the natural negative zeta-potential of EVs [102,103], thus it allows to obtain high purity of the EV fraction, but in a large volume. Hence, it may be combined whit concentration methods, such as dUC or ultrafiltration.

### 3.3. Characterization and Quantification of hAFS-EVs

Reliable techniques for quantification and characterization of EVs are still lacking, thus representing a controversial aspect. To productively translate small hAFS-EVs into advanced medicinal therapy products, it is mandatory that their characterization and quantification may be assessed as reproducible and consistent. MISEV2018 guidelines have suggested using two different, yet complementary, techniques to properly characterise and quantify EVs, trying to avoid any experimental or technical bias [33]. Measurable metrics characterising EV preparations should also inform on the parental cell phenotype, and on the physical and biochemical integrity of the vesicles, according to [104]. Here, we will briefly comment on the methods most commonly use.

#### 3.3.1. Protein Quantification of Small EVs

Protein concentration is commonly used to quantify EVs obtained from stem cell and progenitor cell-conditioned medium. Generally, biochemical assays (i.e., bicinchoninic acid assay or Bradford assay) are commonly used to assess the content of proteins on EV surface. This method is frequently used to assess EV dose for further downstream analysis use in vitro and in vivo; due to the risk of contamination from soluble proteins within the cell secretome, this method may result in high variability and low reproducibility of the experiments. This may be further affected by the different EV isolation method, which influences the amount of the co-precipitating protein. This may explain the very different range of doses used by independent groups in their experimental setting upfront the similar regenerative results obtained (such as Zavatti et al. using 100 µg of hAFSC-EVs in vivo with follow up administrations every 10 days in their osteoarthritis rat model [58], over Balbi et al. using a single administration of 4.5 µg in a preclinical mouse model of myocardial infarction [49]). Small EV canonical biomarkers, such as the tetraspanin protein CD9, CD63, CD81 and syntenin-1, should be also assessed by western blot analysis to validate EV preparation, according to MISEV2018 guidelines [33,105]. This technique may be also indicated to evaluate the relative ratio of parental cell surface antigens (i.e., canonical MSC phenotype based on CD73, CD90, CD105 expression) as an indication of the relative concentration of sEVs in the samples [37].

#### 3.3.2. Evaluation of Number of Particles and Size Distribution in Small EV Preparations

Several methods used can be applied to evaluate the number of particles in EV preparation. One is represented by standard flow cytometry for large EVs [106,107,108,109] and high-resolution flow cytometry for small EVs [110]. The first flow cytometry method is based on magnetic/latex beads binding EVs in combination with lipophilic dyes or with specific antibodies against EV surface antigens (i.e., tetraspanin proteins) [111,112], in order to increase EV dimension in order to detect them with the analyser. High-resolution flow cytometry applies more sophisticated techniques by exploiting appropriate instrumentations, antibodies and controls. An optimised method to specifically evaluate EV particle number is based on nanoparticle tracking analysis (NTA), which is the most common technique to define EV concentration within a sample. NTA is a technique tracking micro- and nano-sized elements based on their Brownian motion of the particle with light scattering [113]. Thus, this method determines the size distribution and also the amount present per ml of EV solution; this technique should be used to express EV concentration and yield as the number of particles (within the 50–200 nm size range) per unit weight protein/membrane lipids to assess the purity of EV preparation over the risk of having contaminating lipids, proteins and nucleic acids [37]. This method may overestimate the particle populations since lipoprotein and protein aggregates are also measured and registered [33]. A solution may rely on using fluorescently conjugated antibodies addressing EV-antigens which could be visualised by an NTA device [114]. Transmission electron microscopy (TEM) imaging may offer a straightforward, yet time-consuming and relatively costly method to visualise EV morphology within a sample while assessing the quality of preparation [91]. Electron microscopy represents the method most commonly adopted to visualise and analyse the morphology of hAFS-EVs. Indeed, some studies have characterised EV size distribution by using TEM in combination with NTA [19,47,67,89], while others have used only TEM techniques [54,87] or just NTA [60]. The comparison of EV size distribution in all these studies suggests that hAFS-EVs cover quite a heterogeneous broad range of distribution. Pierro et al. described two different populations, one ranging around 100 nm and the other one around 200 nm, isolated by dUC for 16h [69]; Takov et al. confirmed the presence of hAFS-EVs of about 110 nm obtained by SEC [19]; Mellows et al. showed an enrichment of hAFSC-EVs between 30–240 nm, with a peak at 90–109 nm, by using fixed-angle rotors in dUC [54]; Balbi et al., among the very first to characterise hAFSC-EVs as separated from hAFSC-CM by dUC for 2 h, described them as ranging from 50 to 600 nm [47,89]; Beretti et al. [87] showed a high enrichment of hAFS-EVs with a size of 35 nm, as obtained by TEIR.

While NTA and TEM have been considered for a long time as the gold standard technique, advanced imaging flow cytometry (IFCM) has been recently developed as improved method allowing single EV analysis directly without the need of any elaborated pre-processing step. Indeed, IFCM may significantly increase the possibility to assess EV heterogeneity in a more reliable, rigorous, and reproducible manner, in order to facilitate the identification of specific subsets of small EVs within the stem/progenitor cell secretome [115].

### 3.4. Definition of Mechanism(s) of Action and Potency Assays

Based on several pre-clinical models, small hAFS-EV preparations have been reported to exert promising effects suggesting their potential as future therapeutics. Nevertheless, a clear understanding of the specific spatio-temporal dynamics and the detailed mechanism of action(s) underlying these positive effects is still lacking. Indeed, most of the works suggest a cause-and-effect link between the hAFS-EV biological activity and the beneficial results obtained in a specific disease setting, due to the potential molecular candidate(s) identified within the EV cargo. Within such a scenario, it is quite difficult at present to establish whether hAFS-EVs may exert different pro-resolving effects (i.e., pro-survival or proliferative influence over immunomodulation) due to independent specific responses to the microenvironment or because they are separated and concentrated from different starting conditions, impacting on their cargo. Several studies seem to pinpoint the hAFS-EV regenerative paracrine effects in their miRNA content, although very heterogeneous and highly variable. Others have reported the proteomic characterization of the hAFS-EV cargo indicating enrichment of a variety of chemokine, cytokine and some specific proteins, such as MIF (macrophage migration inhibitory factor) and SDF-1α (stromal cell-derived factor-1α) [19,89,116], BDNF (brain-derived neurotrophic factor) [56,89], others report the characteristic presence of SOD1 (superoxide dismutase 1) [57], HGF (hepatocyte growth factor), TGFβ (transforming growth factor-β), IDO (indoleamine-pyrrole 2,3-dioxygenase) [58], EMMPRIN (extracellular matrix metalloproteinase inducer) and Agrin [89]. Moreover, it has been reported that hAFS-EVs can carry the *hTERT* gene, an important mediator for organ regeneration induction [117]. While most of the studies discussed here have indicated some putative candidate mechanism of action for the hAFS-EVs regenerative response they described, functional validation is often missing and should be provided. To validate the molecular candidate for a specific therapeutic effect, a common strategy is to demonstrate a direct cause and effect by loss-of-function experiments in which the specific candidate is either silenced, inhibited or blocked in activating their downstream pathways [37]. Moreover, in order to carefully characterise the EV content and investigate their putative mechanism of action, it is also necessary to demonstrate that the candidate protein or genetic sequence of interest is indeed encapsulated and present within the EVs, to avoid any bias caused by their random co-isolation. Hence, it is suggested to treat EV samples with protease, DNase or RNase agent prior to characterising their content [33].

In light of these considerations, there is a strong need for ad hoc potency assays for hAFS-EVs, as comprehensively discussed for MSC-EVs in [37], before considering their translational into the clinics as future therapeutics. A potency assay is a quantitative assay to properly determine the biological activity of the product attribute linked to the relevant biological properties [37]; therefore, it can offer a reliable indication of the mechanism of action of that specific biological element. From the current literature, potency assays for small hAFS-EVs are very likely to vary for different diseases (ischemic-, inflammatory-, chronic-related, etc.) or they can also diverge in similar pathological outcomes (i.e., extension of infarct size, etc.). Moreover, several studies have provided completely different dosing references for hAFS-EV administration in their experimental preclinical models: from hAFS-EV dosage expressed in weight (i.e., µg of hAFS-EVs measured by BCA assay) [47,49,57,58,60,87], to distinct particle numbers or particle number normalised to the specific amount of parental secreting cells (as evaluated by NTA) [19], or as a volume proportion related to the starting cell-condition medium [54,55,67]. This is mainly due to the lack of standardization in the isolating protocols and the analytical procedures for hAFS-EV preparation. This is very likely to affect the EV enrichment with different biological components, thus driving different biological effects in a dose-dependent manner, as also suggested by Antounians et al. [97].

## 4. EVs Derived from Amniotic Fluid: Diagnostics and/or Theranostics?

As previously well described, EVs can be isolated from cell-conditioned medium, as well as from several body fluids (i.e., plasma, serum, synovial fluid, cerebrospinal fluid, seminal fluid, etc.) [30,31]. From this perspective human amniotic fluid (AF) as well can be considered a good source to isolate EVs (hAF-EVs) from it, as informative diagnostic tools with also possible therapeutic profile, hence with *theranostic* potential. hAF-EVs have been described to be relatively small in size (around 100 nm) with the expression of canonical exosomal markers [61]. hAF-EVs have been exploited to provide diagnostic biomarkers via liquid biopsy: their miRNA profile can be informative of specific foetal alteration and disease, such as in the case of severe congenital diaphragmatic hernia [118] or foetal alcohol syndrome [63]. Likewise, the presence of inflammatory factors in hAF-EVs has been shown to correlate with preterm labour [119,120], while other studies have reported hAF-EVs with procoagulant potential.

Notably, hAF-EVs have also been recently described to be able to deliver therapeutic effects. Indeed, EVs concentrated from healthy full-term human amniotic fluid collected during the C-section procedure were shown to preserve alveolar development, antagonise pulmonary hypertension and quench local inflammation in an experimental rat neonatal model of bronchopulmonary dysplasia [64]. Moreover, hAF-EVs have been suggested to exert beneficial effects for spermatogenesis, since enhancing sperm quality in a rat model of azoospermia [121]. Notably, a case report has been recently published, demonstrating for the first time that the systemic administration of a clinically-compliant formulation of AF-EVs (Zofin) was safe, feasible and potentially and therapeutically effective to improve the recovery from complications induced by COVID-19 infection in three different patients [122].

Therefore, hAF-EVs may offer a valuable diagnostic and possibly therapeutic tool to be further exploited in either personalised and/or regenerative medicine. Although the detailed profiling of hAF-EVs along with proof of their functional mechanism of action have not been provided yet, they may represent an appealing source for the future.

## 5. Challenges and Open Questions to Address

**Figure 1:**
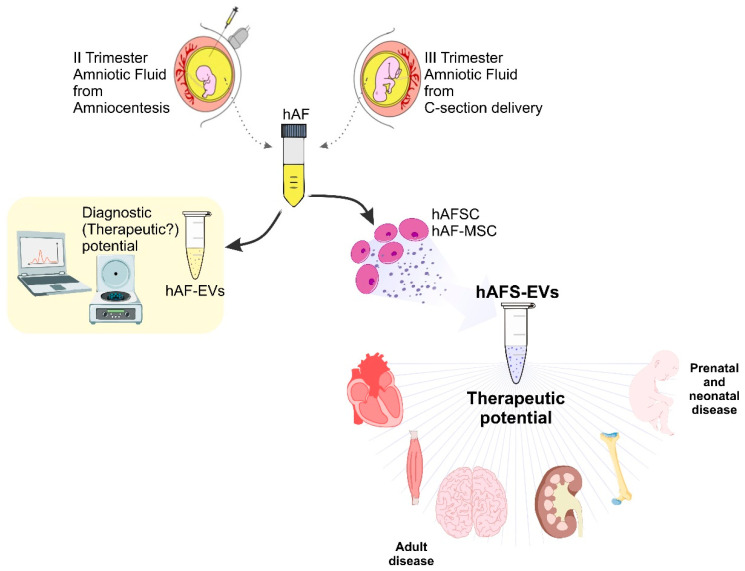
Schematic of the therapeutic and diagnostic potential of hAFSC-EVs and hAF-EVs for future paracrine therapy and personalised medicine. C-section: caesarean-section; hAF: human amniotic fluid; hAF-EVs: human amniotic fluid-derived extracellular vesicles; hAFSC: human amniotic fluid-derived stem cells; hAF-MSC: human amniotic fluid-derived mesenchymal stromal cells; hAFS-EVs: human amniotic fluid stem cell-extracellular vesicles. Schematic has been produced adapting images from Smart—Servier Medical Art (https://smart.servier.com; accessed on 14 December 2021).

Several independent preclinical studies have highlighted the hAFS-EVs remarkable potential to be translated into the clinics as an advanced medicinal therapy product for future paracrine therapy of a variety of diseases; likewise, hAF-EVs may also represent interesting diagnostic and/or theranostics tools to be envisioned in the future, as summarised in Figure 1.

Yet, to date, there is no general consensus on the ideal EV isolation method and on the optimal dose, administration regime and delivery route to use. In addition, ad hoc potency assays should also be provided to validate the hAFS-EV functional mechanism of action as a promising cell-free pharmaceutical-like formulation. Therefore, there is an urgent need for appropriate standardization of manufacturing procedures fulfilling GMP requirements. From this perspective, there are still some open questions to be addressed.

In order to ensure future scaled-up production of hAFS-EVs/hAF-EVs as biotherapeutic agents, it is necessary to discuss volume reducing and purification methods as well as the influence of inter-donor variations on EV profile, as for other MSC-EVs [101]. Moreover, most of the studies have focused on characterising hAFS-EVs from II trimester foetal human amniotic fluid samples obtained during routine prenatal screening (i.e., amniocentesis). Given that the gestational stage may represent a variable influencing the biological profile of hAFS-EVs, Costa et al. recently reported that EVs obtained by II trimester (foetal) hAFSC over III trimester (perinatal) hAFSC (from scheduled Caesarean-section delivery procedures at term) have different profiles in term of the protein content of the EV cargo, thus suggesting a different paracrine potential; notably, foetal and perinatal hAFSC-EVs shared expression of a stable miRNA core [89]. These results are in agreement with independent miRNA analyses from other works [47,54,56], suggesting that small RNA content within hAFSC-EVs may not be influenced by the gestational stage and developmental maturation. Nevertheless, further studies are needed to better identify foetal hAFSC over perinatal ones, as the most suitable source of EVs for paracrine therapy of a certain disease.

Finally, providing the least invasive and safe delivery route (i.e., intravenously), while at the same time ensuring targeting of hAFSC-EVs to the specific tissue/organ to treat, still represents a major challenge for future stem cell/progenitor cell-based paracrine therapy. Since paracrine effects are characterised by very prompt and limited-in-time effects, follow-up administration of hAFSC-EVs may be feasible by means of systemic administration. Therefore, ad hoc engineering of EVs to implement tissue homing and avoid off-target effects should be developed and validated before their clinical translation.

## Figures and Tables

**Table 1:** Comparison of EV separation and concentration techniques.

Separation Method	Advantages	Limits and Concerns
dUC	Good yield Concentration in small volume	Time consuming Protein contamination Heterogeneous EV distribution Costly and specific equipment
Density gradient UC	High specificity for EV fractions High EV purity Concentration in small volume	Time consuming Gradient solution contamination Costly equipment
Ultrafiltration	High reproducibility Less time-consuming	Protein contamination Concern on EV morphology
PEG	Quick procedure User-friendly method Less cost-effective No need for specific equipment	High risk of protein contamination
SEC	High EV functionality High purity of EV fractions High reproducibility Very low protein contamination	Costly reagents Time consuming It may require previous UC step Large end volume of sample to be further concentrated
AEX	High Efficiency High reproducibility Scalability Low protein contamination High EV purity	Large end volume of sample to be further concentrated Specific equipment

dUC: differential ultracentrifugation; UC: ultracentrifugation; PEG: poly-ethylene glycol; SEC: size-exclusion chromatography; AEX: anion-exchange chromatography.
